# Encapsulating TiO_2_ Nanoparticles in Chitosan-Based Porous Microspheres as Multifunctional UV Filters

**DOI:** 10.1021/acs.langmuir.6c01320

**Published:** 2026-06-05

**Authors:** Yunxing Li, Xue Cai, Beizhe Chang, Tong Yang, Hang Jiang, To Ngai

**Affiliations:** † Key Laboratory of Synthetic and Biological Colloids, Ministry of Education, School of Chemical and Material Engineering, 66374 Jiangnan University, Wuxi 214122, China; ‡ Department of Chemistry, The Chinese University of Hong Kong, Hong Kong 999077, China

## Abstract

Titanium dioxide nanoparticles (TiO_2_ NPs) are widely used as inorganic UV filters, yet their photocatalytic activity and resulting phototoxicity raise serious safety issues. Here, we report a Pickering double emulsion templating strategy for constructing chitosan-based porous microspheres encapsulating TiO_2_ NPs (TiO_2_@CS-PMs), with controllable microstructures and mass loading of TiO_2_ NPs. The oil-in-water-in-oil double emulsion template confines TiO_2_ NPs within a cross-linked porous polymer skeleton. Structural confinement, combined with the antioxidant capacity of chitosan (CS), greatly reduces UV-induced reactive oxygen species generation of TiO_2_ NPs. In vitro assays reveal that TiO_2_@CS-PMs have good biocompatibility and significantly lower phototoxicity to HaCaT cells than naked TiO_2_ NPs. The porous architecture promotes optical confinement, and the encapsulation causes interfacial interactions between TiO_2_ NPs and the CS matrix, resulting in broadened and enhanced UV attenuation. Sunscreen incorporating TiO_2_@CS-PMs exhibits higher sun protection factors and cellular photoprotection at lower TiO_2_ NPs doses, while the CS matrix provides antibacterial activity. This work reveals Pickering double emulsion templating porous microstructure as a versatile platform for managing particle photochemistry, providing insights into the development of safer and multifunctional UV filters.

## Graphical abstract

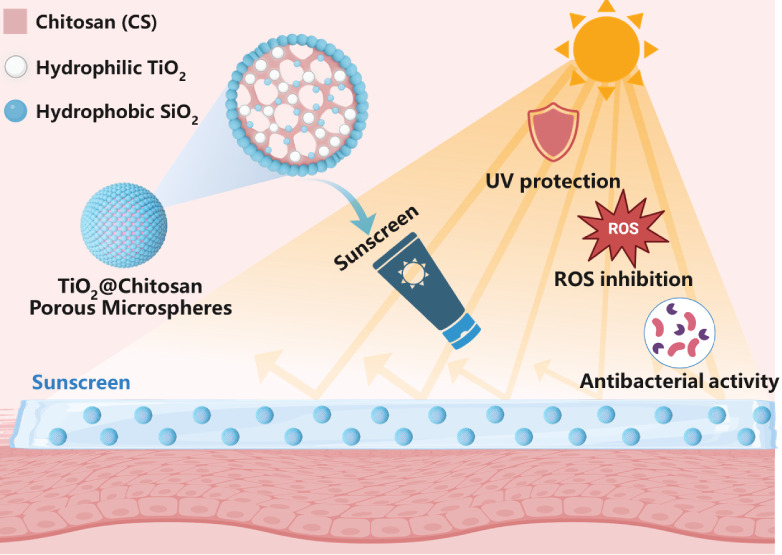

## 1 Introduction

Prolonged ultraviolet (UV) exposure causes photoaging, erythema, and an increased risk of skin cancer.^1–4^ Sunscreens are used daily to prevent UV-induced skin damage, and their efficacy is heavily dependent on UV filters that block harmful radiation. UV filters are broadly classified into organic and inorganic categories. Inorganic UV filters generally provide superior photostability, broad-spectrum coverage, and limited skin penetration, making them indispensable in modern sunscreen formulations. Titanium dioxide (TiO_2_) and zinc oxide nanoparticles (NPs) are the most common inorganic UV filters. In particular, TiO_2_ NPs have attracted extensive attention because of their strong UV attenuation, high stability, and commercial availability.^5–7^ However, under UV irradiation, inorganic UV filters, particularly TiO_2_ NPs, can generate reactive oxygen species (ROS) due to their photocatalytic nature that can cause skin irritation, accelerate photoaging, and exacerbate the breakdown of organic ingredients in sunscreens.^8–10^ Therefore, effective ROS suppression is essential for the development and safe use of sunscreens containing TiO_2_ NPs.

To address the photocatalytic reactivity of TiO_2_ NPs, several strategies have been devised to control their interaction with the surrounding environment. Surface coating is a typical method for physically blocking reactive sites and limiting the photogenerated ROS diffusion.^11–15^ However, establishing uniform and continuous conformal coatings on nanoscale particles is still technically challenging, and even minor coating imperfections can compromise photocatalytic suppression.^16–18^ Furthermore, discharged nanoscale particles are difficult to collect, raising concerns regarding their long-term environmental impact. Alternatively, encapsulation strategies that immobilize inorganic UV filters within micro- or submicroscale carriers have attracted increasing attention,^19–22^ since physical isolation from the external environment can more reliably block photocatalytic reactions while improving handling and recovery.

Spherical porous materials are especially appealing encapsulation carriers because their hierarchical architectures can improve UV attenuation through multiple light scattering and reflection.^23,24^ For example, Nam et al. reported the layer-by-layer assembly of tannic acid (TA) and TiO_2_ NPs on porous poly­(methyl methacrylate) microspheres, in which the porosity design increased UV-shielding performance, and the polyphenol layer substantially suppressed the photochemical ROS generation from TiO_2_ NPs.^17^ Following that, a subsequent work refined the encapsulation system by using micrometer-sized mesoporous SiO_2_ particles (MSN) as a template to construct a multi-interfacial SiO_2_–TiO_2_–polyphenol heterojunction, resulting in efficient photocatalytic suppression and a plastic-free carrier.^25^ Zhang et al. reported a similar process in which TiO_2_ NPs were loaded into MSN and further sealed with a SiO_2_ shell to form an MSN-TiO_2_@SiO_2_ composite structure.^26^

Despite their effectiveness, these systems often require multiple steps to fabricate porous carriers, with the immobilization of TiO_2_ NPs performed in a subsequent and separate step, complicating processing and limiting scalability. Furthermore, they are primarily made of inorganics or synthetic polymers, which have low biocompatibility and poor biodegradability, thus limiting their sustainability and safety requirements for topical applications.^27–29^ Natural polymers, on the other hand, have emerged as intriguing alternatives owing to their renewable nature and attractive biological properties.^30^ Chitosan, a naturally derived cationic polysaccharide, combines nontoxicity, biodegradability, antioxidant, and antibacterial activity, making it an attractive material for constructing functional porous carriers with inherent biological benefits.^31,32^

In this study, we present a simple and scalable encapsulation strategy that employs oil-in-water-in-oil (O/W/O) Pickering double emulsions as templates to fabricate chitosan-based porous microspheres loaded with TiO_2_ NPs (TiO_2_@CS-PMs). Notably, the formation of porous microspheres and the immobilization of TiO_2_ NPs were accomplished simultaneously via a one-step emulsification followed by in situ cross-linking. The structural characteristics, chemical composition, and photoprotective and biological performance of the resultant microspheres were systematically examined to assess their suitability for topical UV protection. This work proposes a simple and sustainable strategy for developing multifunctional UV filters that combine effective UV shielding with improved biosafety and additional biological functionality.

## 2 Experimental Section

### 2.1 Materials

Chitosan (CS) and fluorescein isothiocyanate (FITC) were supplied by Sigma-Aldrich (USA). Genipin and potassium persulfate were obtained from Aladdin (Shanghai, China). Rhodamine B (RhB), perylene, titanium dioxide nanoparticles (TiO_2_ NPs), 2,2-diphenyl-1-picrylhydrazyl (DPPH), methylene blue (MB), 2,2’-azino-bis (3-ethylbenzthiazoline-6-sulfonic acid) (ABTS), and methyl orange (MO) were purchased from Macklin (China). Hydrophobic AEROSIL R974 (SiO_2_ NPs) was provided by Evonik (Germany). Cyclopentasiloxane (D5) was purchased from Keyin Industrial Co., Ltd. (Shanghai, China). Zinc oxide (ZnO) was kindly provided by Hope-Tec (Shanghai, China). A commercial cream was supplied by Nivea (Germany). HaCaT cells were purchased from the BeNa Culture Collection (Beijing, China). Dulbecco’s modified Eagle medium (DMEM), fetal bovine serum (FBS), and phosphate-buffered saline (PBS) were supplied by Hyclone (USA). Cell Counting Kit-8 (CCK-8), Calcein-AM, and propidium iodide (PI) were acquired from Beyotime (Shanghai, China). 2’,7’-dichlorofluorescin-diacetate (DCFH-DA) was supplied by Adamas-life (China). Sodium chloride, agar, tryptone, and yeast extract were sourced from Sinopharm Chemical Reagent Co., Ltd. (China) and Thermo Fisher Scientific (USA), respectively.

### 2.2 Preparation of TiO_2_@Chitosan Porous Microspheres (TiO_2_@CS-PMs)

CS (3%, w/v) was dissolved in aqueous acetic acid (2 vol %) under magnetic stirring. TiO_2_ NPs were subsequently added at various masses (30, 90, and 150 mg), corresponding to final concentrations of 1%, 3%, and 5% (w/v) in the CS solution. The mixture was stirred until a homogeneous dispersion was obtained. In parallel, SiO_2_ NPs (1%, w/v) were dispersed in D5 by ultrasonication to prepare the oil phase. Genipin solution (12 mg/mL) was then introduced into the CS solution containing TiO_2_ NPs, briefly vortexed, and stirred to ensure uniform distribution. Immediately after genipin addition, the aqueous phase was mixed with an equal volume of as-prepared oil phase and emulsified by a cell disruptor (390 W, 2 min) to generate O/W/O Pickering double emulsions. After in situ cross-linking of CS with genipin, the resulting porous microspheres were washed and subsequently freeze-dried. The obtained samples were denoted as CS-PMs (without TiO_2_ NPs) and T_1_@CS-PMs, T_3_@CS-PMs, and T_5_@CS-PMs according to the amount of TiO_2_ NPs added.

To investigate the influence of oil/water (O/W) volume ratio and CS concentration, additional samples were prepared by varying the CS concentration (2.0 and 2.5%) or the O/W volume ratio (1:2 and 2:1), while keeping other parameters constant. Nonporous CS microspheres were prepared as the control by emulsifying CS solution (3%, w/v) containing TiO_2_ NPs (1%, w/v) at an O/W ratio of 4:1, followed by identical cross-linking and post-treatment steps, and denoted as CS-Ms.

### 2.3 Characterization

The morphology and elemental distribution of TiO_2_@CS-PMs were characterized by a Hitachi S-4800 scanning electron microscope (SEM, Japan) equipped with energy-dispersive spectroscopy (EDS) and a JEOL JEM-2100 Plus transmission electron microscope (TEM, Japan). Confocal laser scanning microscopy (CLSM) images were obtained using a Nikon AX microscope (Japan) with excitation wavelengths of 405, 488, and 561 nm. Fourier-transform infrared (FTIR) spectra were acquired using a Nicolet iS50 FTIR spectrometer (Thermo Fisher Scientific, USA) with the KBr pellet method over a range of 500 to 4000 cm^–1^. X-ray diffraction (XRD) patterns were recorded using a Bruker AXS D8 diffractometer (Germany) with Cu Kα radiation. Thermogravimetric analysis (TGA) was conducted using a Mettler Toledo 1100SF instrument (Switzerland) under an oxygen atmosphere at a heating rate of 20 °C/min. UV–vis transmittance spectra were recorded using a Shimadzu UV-3600 Plus spectrophotometer (Japan) from 200 to 400 nm.

### 2.4 Evaluation of Photocatalytic Activity

TiO_2_ NPs (0.1 mg/mL), T_1_@CS-PMs (0.5 mg/mL), CS-PMs (0.5 mg/mL), or a physical mixture of TiO_2_ NPs (0.1 mg/mL) and CS-PMs (0.4 mg/mL) were dispersed in MB aqueous solution (0.01 mg/mL) by ultrasonication. Sample concentrations were selected to ensure comparable TiO_2_ NPs or carrier content. The dispersions were irradiated under UV light (365 nm, 80 W) for 1 h. Aliquots were withdrawn every 20 min, centrifuged to remove suspended particles, and the absorbance of the supernatant at 664 nm was measured. The residual MB percentage at time *t* (*C*_t_) was determined from the absorbance and normalized to the initial concentration (*C*_0_) to evaluate photocatalytic degradation.

### 2.5 Antioxidant Performance of TiO_2_@CS-PMs

T_1_@CS-PMs were first diluted in absolute ethanol to various concentrations (0.625 to 5 mg/mL). For the DPPH• assay, equal volumes of sample dispersion and DPPH• solution (0.05 mg/mL) were mixed and incubated for 30 min in the dark. Then, the combination was centrifuged, and the absorbance (517 nm) of the supernatant was measured. The scavenging rate was calculated as follows:

$$\mathrm{DPPH• scavenging rate}\left(\%\right){=[(A}_{\mathrm{DPPH•}}-A_{s}{)/A}_{\mathrm{DPPH•}}]\times 100\%$$

where *A*_DPPH*•*_ and *A*_*s*_ are the absorbance of the DPPH• solution before and after reaction with T_1_@CS-PMs.

For the ABTS assay, a working solution was obtained by mixing ABTS (7 mM) with potassium persulfate (2.46 mM) for 12 h. Equal volumes of sample dispersion and working solution were mixed and incubated for 30 min in the dark, followed by the measurement of absorbance (734 nm). The scavenging rate was determined with the following formula:

$${\mathrm{ABTS}}^{+}•\mathrm{scavenging rate}\left(\%\right)=[(A_{{\mathrm{ABTS}}^{+}•}-A_{s})/A_{{\mathrm{ABTS}}^{+}•}]\times 100\%$$

where *A*_ABTS+•_ and *A*_*s*_ denote the absorbance of the ABTS^+^• solution before and after incubation with T_1_@CS-PMs.

### 2.6 Cytotoxicity Evaluation with or without UV Irradiation

HaCaT cells were cultured in a modified DMEM containing penicillin–streptomycin (1 vol %) and FBS (10 vol %). Then, cells were seeded in 96-well plates (1 × 10^4^ cells per well) and cultured for 24 h. Following that, cells were treated with 100 μL of DMEM containing T_1_@CS-PMs at different concentrations (12.5 to 100 μg/mL). After 24 h of incubation, the cytotoxicity was evaluated using the CCK-8 assay. Six replicates were used for the experiment. To assess photoinduced cytotoxicity, HaCaT cells were seeded into 12-well plates (3 × 10^5^ cells per well) and cultured for 12 h before being treated with T_1_@CS-PMs or TiO_2_ NPs for 1 h. Both samples included an equal amount of TiO_2_ NPs (10 μg/mL). Afterward, the cells were subjected to UV exposure (311 nm, 40 W) for 5 min, followed by a further 12 h of culture. CCK-8 viability and live/dead double-staining (Calcein AM and PI) assays were performed. DCFH-DA staining and CLSM imaging were used to assess intracellular ROS levels under identical irradiation and treatment settings.

### 2.7 Evaluation of UV-Shielding Performance

Sunscreens were prepared with a Nivea cream as the base matrix. Unless otherwise specified, each formulation included ZnO (10 wt %) and an additional 5 wt % of TiO_2_ NPs, CS-PMs, CS-Ms, T_1_@CS-PMs, T_3_@CS-PMs, or T_5_@CS-PMs. The corresponding formulations were denoted as TiO_2_ Sunscreen, CS-PMs Sunscreen, CS-Ms Sunscreen, T_1_@CS-PMs Sunscreen, T_3_@CS-PMs Sunscreen, and T_5_@CS-PMs Sunscreen, respectively. For comparison, a control formulation containing only ZnO (10 wt %) was prepared and denoted as the control group. All formulations were prepared by continuously stirring at 60 °C to evenly disperse the sample particles in the base matrix. A UV-2000s transmittance analyzer (Labsphere, USA) was used to assess the in vitro sun protection factor (SPF). Sunscreens (50 mg) were evenly distributed on PMMA plates (50 × 50 mm) and dried in the dark for 15 min. The SPF values were obtained by measuring 12 random points per plate, with five replicate plates per sample. The photostability of T_1_@CS-PMs Sunscreen was investigated under simulated solar light using a PLS-SXE300+/UV lamp (PerfectLight, China). The SPF values were periodically measured after 2, 4, 6, and 8 h of irradiation to analyze the photostability based on the variation of SPF values.

For the MO degradation experiment, an MO solution (0.01 mg/mL) was prepared, and TiO_2_ NPs were added to achieve a final concentration of 0.05 mg/mL. After stirring in the dark for 30 min to achieve adsorption equilibrium, 15 mL of the mixture was transferred to a series of Petri dishes. UV shielding was achieved using Petri dish lids (90 × 15 mm) coated with various sunscreens (2 mg/cm^2^). These samples were vertically irradiated using a UV light (311 nm, 40 W). At regular intervals, aliquots were taken, centrifuged, and the absorbance of MO solution at 465 nm was measured. All experiments were done in triplicate.

To test the cellular protection of various sunscreens, HaCaT cells were seeded into 12-well plates with 3 × 10^5^ cells per well and incubated for 12 h. After washing with PBS, various sunscreens were evenly applied to the surfaces of plate lids (2 mg/cm^2^) to serve as UV-filtering barriers. The cells were then subjected to UV irradiation (311 nm, 40 W) for 5 min through the coated lids, followed by a further 12 h of incubation. Cell viability was measured using the CCK-8 assay kit, and cell survival was determined by live/dead staining with Calcein AM and PI, followed by CLSM observation.

### 2.8 Antibacterial Activity Assay of TiO_2_@CS-PMs

Antibacterial activity against *Escherichia coli* (*E. coli*) and *Staphylococcus aureus* (*S. aureus*) was evaluated using the plate count method. Bacteria were cultured in Luria–Bertani broth until logarithmic growth phase at 37 °C, then diluted to 1 × 10^6^ CFU/mL. Bacterial suspensions were placed in 12-well plates and cultured with 250 μL of T_1_@CS-PMs or CS-PMs at various doses for 12 h. The treated samples were serially diluted, spread onto agar plates, and incubated for colony counting. For SEM analysis, bacteria were recovered by centrifugation, washed with PBS, and fixed in 2.5 wt % glutaraldehyde solution at 4 °C overnight. Fixed bacteria were subsequently dehydrated with a graded ethanol series ranging from 20 to 100 vol %.

### 2.9 Statistical Analysis

All quantitative data were presented as mean ± standard deviation from at three independent experiments. Statistical comparisons among multiple groups were conducted using one-way analysis of variance (ANOVA) followed by Tukey’s post hoc test. Statistical significance was considered at *p < 0.05, **p < 0.01, ***p < 0.001, and ****p < 0.0001.

## 3 Results and Discussion

### 3.1 Preparation and Characterization of TiO_2_@CS-PMs

The fabrication procedure for TiO_2_@CS-PMs is schematically illustrated in Scheme 1. TiO_2_ NPs were homogeneously dispersed in an aqueous CS solution, after which genipin was added as a naturally occurring cross-linker. The resultant aqueous phase was immediately emulsified with an oil phase containing dispersed SiO_2_ NPs in an ultrasonic-assisted one-step procedure. The increased viscosity of the aqueous phase, as well as the phase inversion, is responsible for the preparation of a stable O/W/O Pickering double emulsion.^33,34^ The larger water droplets formed later capture oil droplets during phase inversion. The high viscosity of the CS solution hinders the mobility of trapped oil droplets. Moreover, the steric barrier of SiO_2_ NPs and CS prevents the diffusion of inner oil droplets into the external oil phase. In-situ cross-linking between CS and genipin occurred within the intermediate phase of the double emulsion, resulting in the formation of a three-dimensional CS network that served as the skeleton for the resulting porous microspheres. Simultaneously, TiO_2_ NPs were immobilized within the polymeric framework. The internal oil droplets, which acted as sacrificial templates, were subsequently removed, resulting in TiO_2_@CS-PMs with porous architecture.

**Scheme 1:**
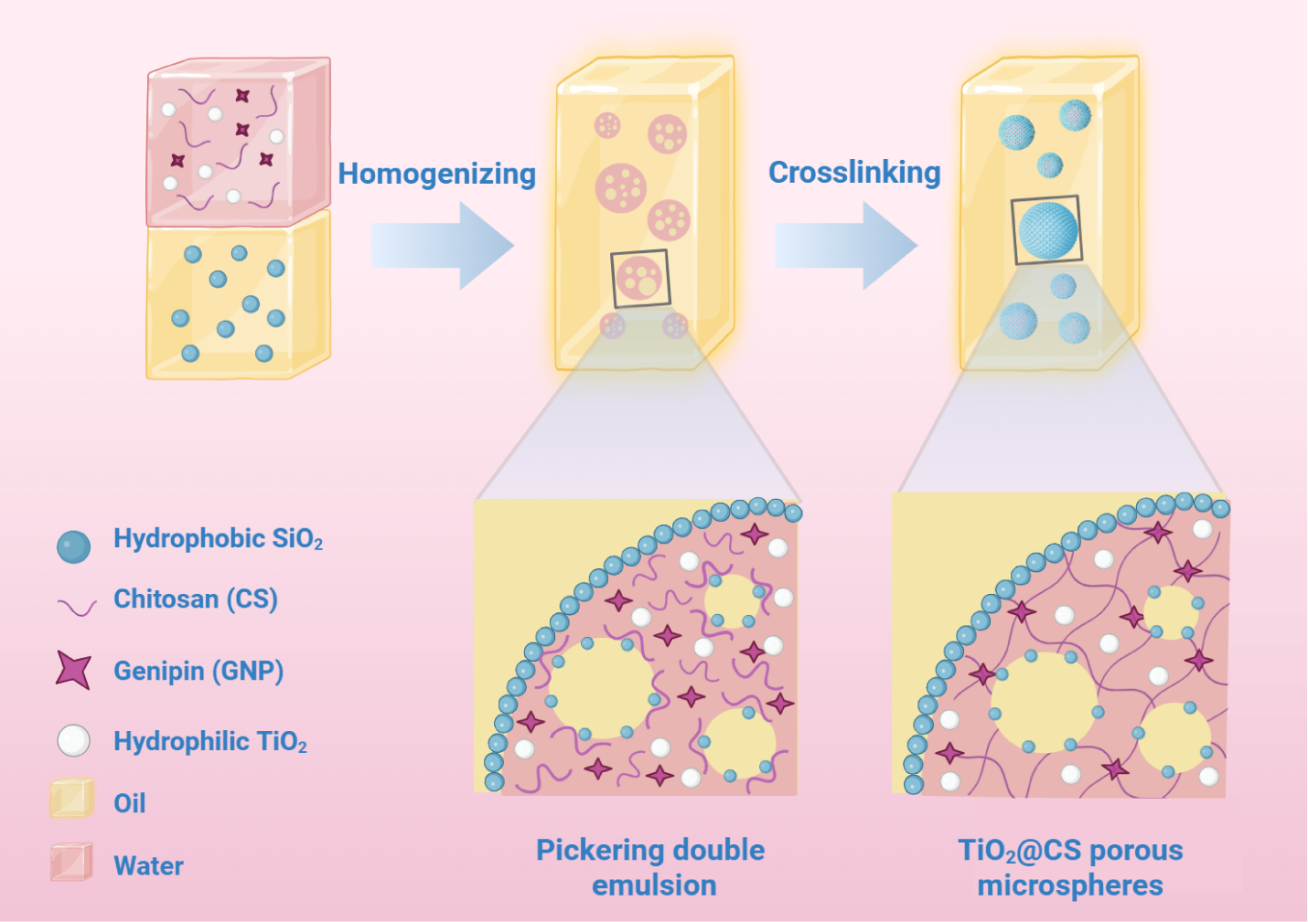
Schematic Illustration of the Fabrication of TiO_2_@CS-PMs Templated from the Pickering Double Emulsion

CLSM was initially used to in situ visualize a freshly prepared double emulsion-templated porous structure generated with 30 mg TiO_2_ NPs (T_1_@CS-PMs) using selective fluorescent labeling to validate their effective preparation and structural characteristics. Green fluorescence was detected in CS tagged with FITC, red fluorescence in SiO_2_ NPs labeled with RhB, and blue fluorescence in the oil phase labeled with perylene.^35^ As presented in Figure 1a, green fluorescence is primarily distributed in the intermediate aqueous phase, indicating that CS serves as the microsphere skeleton, whereas the blue fluorescence detected within the microspheres and in the continuous phase indicates that encapsulated inner oil droplets form the internal voids. Red fluorescence seen at the oil–water interface confirms the interfacial stabilizing function of SiO_2_ NPs. Three-dimensional CLSM reconstruction (Figure S1) also indicates that the porous architecture is derived directly from the Pickering double emulsion template. The oil/water volume ratio was found to be an important parameter in determining the double emulsion template and the resulting porous architecture (Figures S2 and S3). Increasing the aqueous phase fraction expanded the outer aqueous droplets and inner oil droplets, resulting in larger internal pore sizes and microsphere diameters. These results also demonstrate that the porous structure of TiO_2_@CS-PMs is derived directly from the O/W/O Pickering double emulsion template.

**Figure 1:**
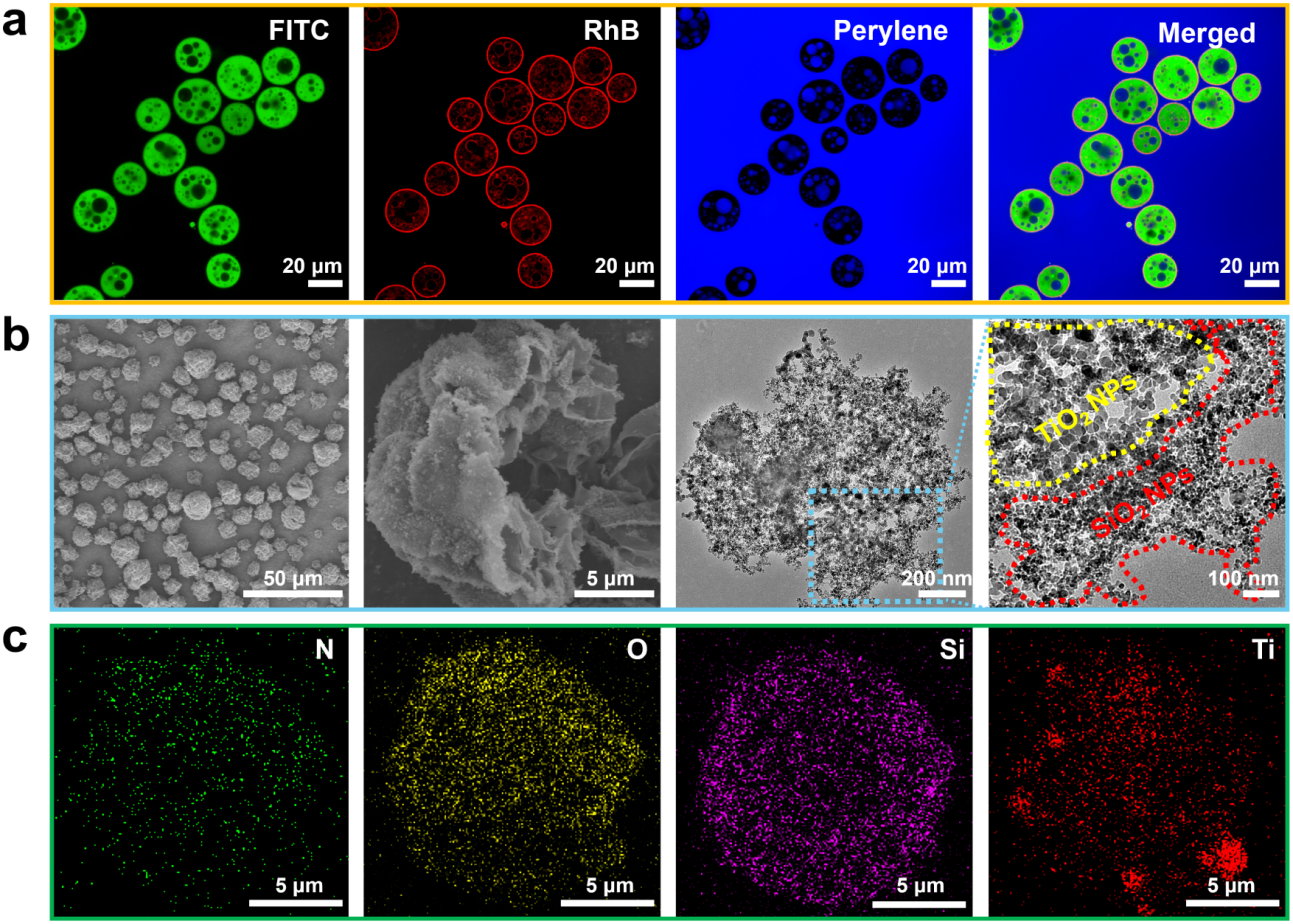
(a) CLSM, (b) SEM, and TEM images as well as (c) corresponding EDS element mapping of T_1_@CS-PMs.

After drying, SEM images (Figure 1b) reveal that T_1_@CS-PMs are well dispersed but have irregular morphologies, which can be attributed to the partial collapse of the polymeric skeleton during the water removal process.^36^ Many NPs attached to the microsphere surface are identified as SiO_2_ NPs. This drying-induced shrinkage is supported further by samples prepared at lower CS concentrations, which have similar emulsion template sizes in the wet state but significantly smaller microsphere sizes after drying, indicating insufficient skeletal strength to fully preserve the templated geometry (Figures S4 and S5). Notably, a higher-magnification image of fractured microspheres shows a highly wrinkled, sponge-like internal framework with micron-scale spaces, indicating a porous interior rather than a dense solid structure, which is consistent with CLSM findings.

Figure S6 shows the TEM images and particle size distributions of pristine TiO_2_ and SiO_2_ NPs, and they exhibited distinct particle sizes, with SiO_2_ NPs (17.40 nm) being smaller than TiO_2_ NPs (24.53 nm). As shown in Figure 1b, there are a considerable number of electron-dense NPs in the prepared microspheres, which correspond to TiO_2_ and SiO_2_ NPs with two distinct size populations. The internal porous structure is not well-defined due to the low electron density of the CS skeleton and the projection nature of TEM, which reduces contrast between voids and the surrounding materials. The larger NPs are primarily distributed within the microsphere interior and are identified as TiO_2_ NPs, whereas the smaller NPs are mainly situated around the microsphere periphery and are identified as SiO_2_ NPs, as further validated by elemental mapping (Figure 1c).^37,38^ Furthermore, elemental mapping reveals a homogeneous yet discrete distribution of nitrogen throughout the microspheres, confirming that CS serves as the structural skeleton of the porous microspheres. This spatial segregation demonstrates the inheritance of polymer and particle localization in the emulsion template, with TiO_2_ NPs remaining in the aqueous phase and becoming fixed inside the cross-linked CS network, whereas SiO_2_ NPs preferentially adsorb at the oil–water boundary. This result is corroborated by contact angle measurements (Figure S7), which show a transition from the hydrophilic surface of TiO_2_ NPs to a hydrophobic surface for T_1_@CS-PMs, consistent with SiO_2_ NPs’ peripheral enrichment and TiO_2_ NPs’ internal encapsulation.

FTIR spectroscopy was employed to verify the chemical composition and cross-linking of the porous microspheres. As shown in Figure 2a, pristine CS has a broad absorption around 3416 cm^–1^ due to partially overlapped stretching vibrations of O–H and N–H, as well as amide I (C=O) and amino group at about 1653 and 1550 cm^–1^, and genipin has characteristic stretching vibrations of C=O and C=C at about 1681 and 1621 cm^–1^, respectively.^39,40^ SiO_2_ NPs display typical Si–O–Si stretching vibrations at about 1107 and 811 cm^–1^, while TiO_2_ NPs show a weak peak at around 1399 cm^–1^ and broad absorption below 1000 cm^–1^ for Ti–O–Ti stretching vibration.^41,42^ In the spectrum of T_1_@CS-PMs, some characteristic bands associated with these components are visible, showing that they are successfully incorporated. Furthermore, the disappearance of the carbonyl stretching band associated with the ester group of genipin, as well as the emergence of an amide II band at about 1559 cm^–1^, indicates the occurrence of genipin-mediated cross-linking.^43^

**Figure 2:**
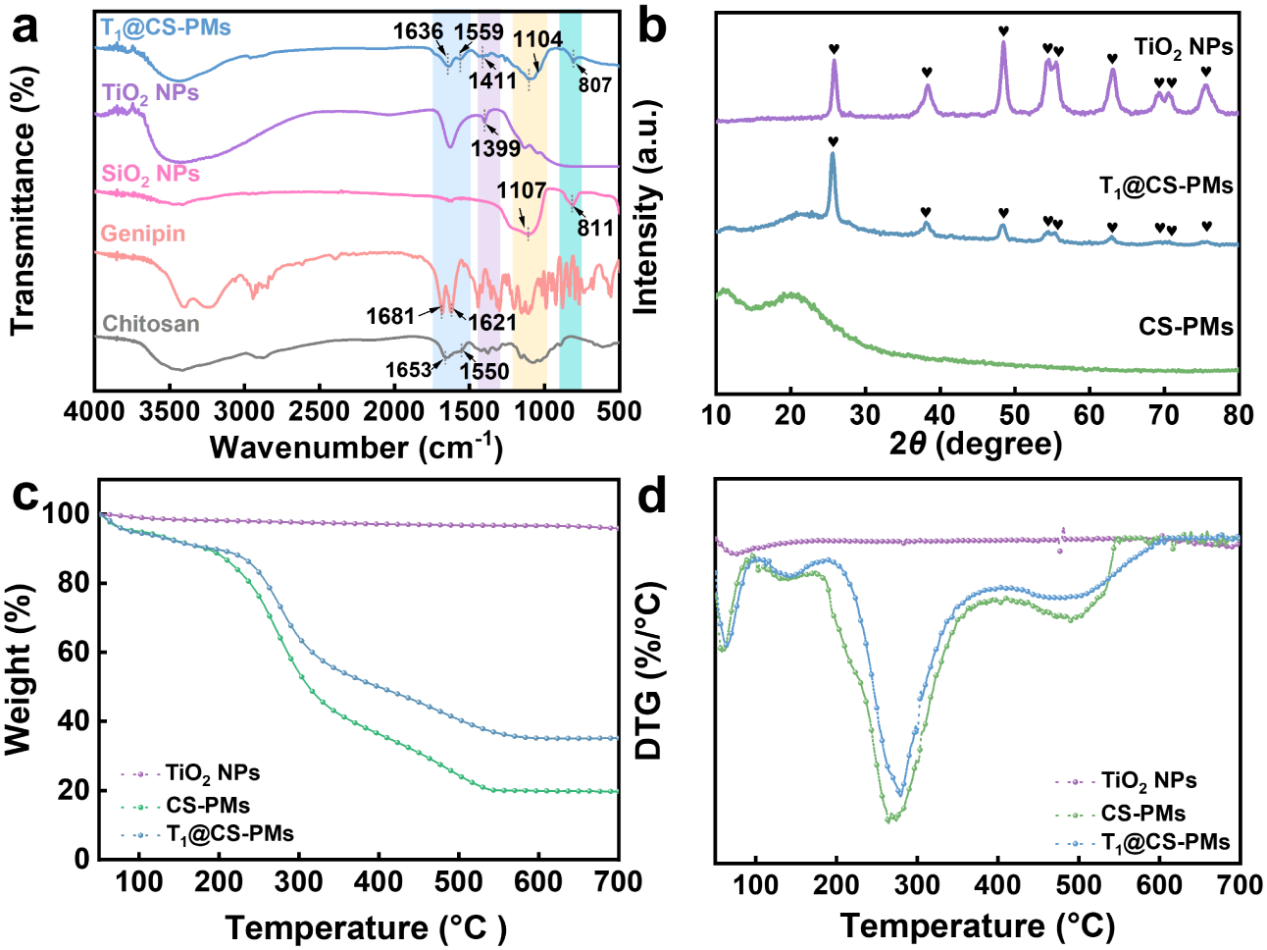
(a) FT-IR spectra of chitosan, genipin, SiO_2_ NPs, TiO_2_ NPs, and T_1_@CS-PMs; (b) XRD patterns as well as (c) TGA and (d) DTG curves of TiO_2_ NPs, T_1_@CS-PMs and CS-PMs.

The phase structures of CS-PMs, TiO_2_ NPs, and T_1_@CS-PMs were further investigated by XRD (Figure 2b). CS-PMs have a broad diffraction halo, indicating that the CS and SiO_2_ NPs are amorphous. In contrast, TiO_2_ NPs display distinct crystalline diffraction peaks at about 25.74°, 38.37°, 48.37°, 53.43°, and 55.04°, which correspond to anatase phase lattice planes of (101), (104), (200), (105), and (211), according to JCPDS card no. 21–1272. The XRD pattern of T_1_@CS-PMs shows the presence of both the amorphous background and the characteristic diffraction of TiO_2_ NPs, indicating the successful incorporation of TiO_2_ NPs into porous microstructures. Furthermore, the constant peak positions demonstrate that the crystal structure of TiO_2_ NPs remains unchanged during emulsification and cross-linking, whereas the lower peak intensity is due to dilution by the amorphous matrix and spatial confinement within the microspheres.

To test the robustness and compositional tunability of the proposed fabrication strategy, a series of porous microspheres were prepared with varied amounts of TiO_2_ NPs. Optical microscopy images (Figure S8) show that increasing the amount of TiO_2_ NPs added gradually increases light attenuation within the water droplets and microspheres, as evidenced by expanded dark patches, indicating efficient incorporation of TiO_2_ NPs into the porous microspheres.^44,45^ In contrast, the average droplet sizes of double emulsion templates and the average diameters of the resulting microspheres do not show a monotonic relationship with the amount of TiO_2_ NPs added, implying that TiO_2_ NPs have no significant influence on the emulsion templating process. After cross-linking, all samples have comparable sizes and irregular morphologies, as revealed by SEM images (Figure S9), which are attributable to drying-induced shrinkage of the polymeric structure. These results demonstrate that the formation of TiO_2_@CS-PMs is structurally robust over a wide concentration range of TiO_2_ NPs.

TGA was subsequently employed to determine the composition tunability of TiO_2_@CS-PMs (Figure 2c). TiO_2_ NPs showed negligible weight loss over temperatures ranging from 25 to 700 °C, indicating high thermal stability. In contrast, CS-PMs exhibited an initial weight loss below 200 °C, which is due to the release of physically adsorbed water and the probable weakening of a part of the CS structure due to cross-linking.^46^ A considerable decomposition occurs between 200 and 300 °C, corresponding to the thermal deterioration of the CS skeleton, followed by weight loss at higher temperatures associated with the disintegration of residual cross-linked polymer chains.^22^ The remaining mass of CS-PMs is mostly composed of thermally stable SiO_2_ NPs. T_1_@CS-PMs degraded similarly to CS-PMs, demonstrating the presence of a polymeric matrix, but with substantially higher residual mass due to the combined contributions of SiO_2_ and TiO_2_ NPs. Notably, as shown in Figure 2d, the DTG curves show that T_1_@CS-PMs have a higher main decomposition temperature (279 °C) than CS-PMs (268 °C). This enhancement is related to the presence of thermally stable TiO_2_ NPs, confirming the successful encapsulation of TiO_2_ NPs and the formation of TiO_2_@CS-PMs. Because the amounts of SiO_2_ NPs are constant in both CS-PMs and T_1_@CS-PMs, the increased residual mass can be attributed to the encapsulated TiO_2_ NPs. Furthermore, TGA data show a gradual increase in residual mass as the amount of TiO_2_ NPs added increases, demonstrating that the content of TiO_2_ NPs in the TiO_2_@CS-PMs could be quantitatively regulated (Figure S10). These results are consistent with those seen in optical microscopy images in Figure S8. The experimentally measured weight percentages of TiO_2_ NPs of T_1_@CS-PMs, T_3_@CS-PMs, and T_5_@CS-PMs are 19.51, 30.48, and 40.47 wt %, respectively, which closely matched the theoretical values and correspond to encapsulation efficiencies of approximately 97.55, 83.27, and 82.59%.

### 3.2 Structural Confinement-Induced Suppression of ROS Generation

Effective suppression of UV-induced ROS formation is a prerequisite for the safe use of TiO_2_ NPs in sunscreen formulations, as photoinduced ROS are associated with oxidative skin damage and inflammatory responses. The photocatalytic degradation of MB, an indirect indicator of ROS production, was used to assess the photochemical reactivity of different samples. Figure 3a shows that MB degradation was negligible in the absence of TiO_2_ NPs and in the presence of CS-PMs. In contrast, naked TiO_2_ NPs rapidly degraded MB to around 8% of its initial concentration within 60 min of UV irradiation, accompanied by nearly complete solution decoloration (Figure S11), demonstrating their significant photoinduced ROS-generating capability. Notably, the MB retention rate increased to 26.32% when a physical mixture of TiO_2_ NPs and CS-PMs was present. More significantly, the addition of T_1_@CS-PMs greatly reduced MB degradation under identical UV exposure, preserving around 80% of MB and retaining a distinct blue color. This substantial passivation finding demonstrates that encapsulation effectively suppresses the photocatalytic activity of TiO_2_ NPs.^26,47^ This suppression is primarily due to structural confinement within the cross-linked CS matrix, which physically isolates TiO_2_ NPs from the external environment and reduces ROS generation and diffusion. In addition, CS has antioxidant activity, which may help with compositional ROS buffering.^31^ Consistently, T_1_@CS-PMs exhibited concentration-dependent radical scavenging activity in both the DPPH• and ABTS^+^• assays (Figure 3b and c). When the concentration of T_1_@CS-PMs was raised to 5 mg/mL, the scavenging efficiency of DPPH• and ABTS^+^• exceeded 90%. This is consistent with the result obtained from the physical mixture of TiO_2_ NPs and CS-PMs, indicating that ROS generated by TiO_2_ NPs under UV irradiation was partially scavenged. These results show that the synergistic effects of structural confinement and compositional antioxidative buffering significantly increase the safety of T_1_@CS-PMs with UV exposure, highlighting their potential as a novel UV filter.

**Figure 3:**
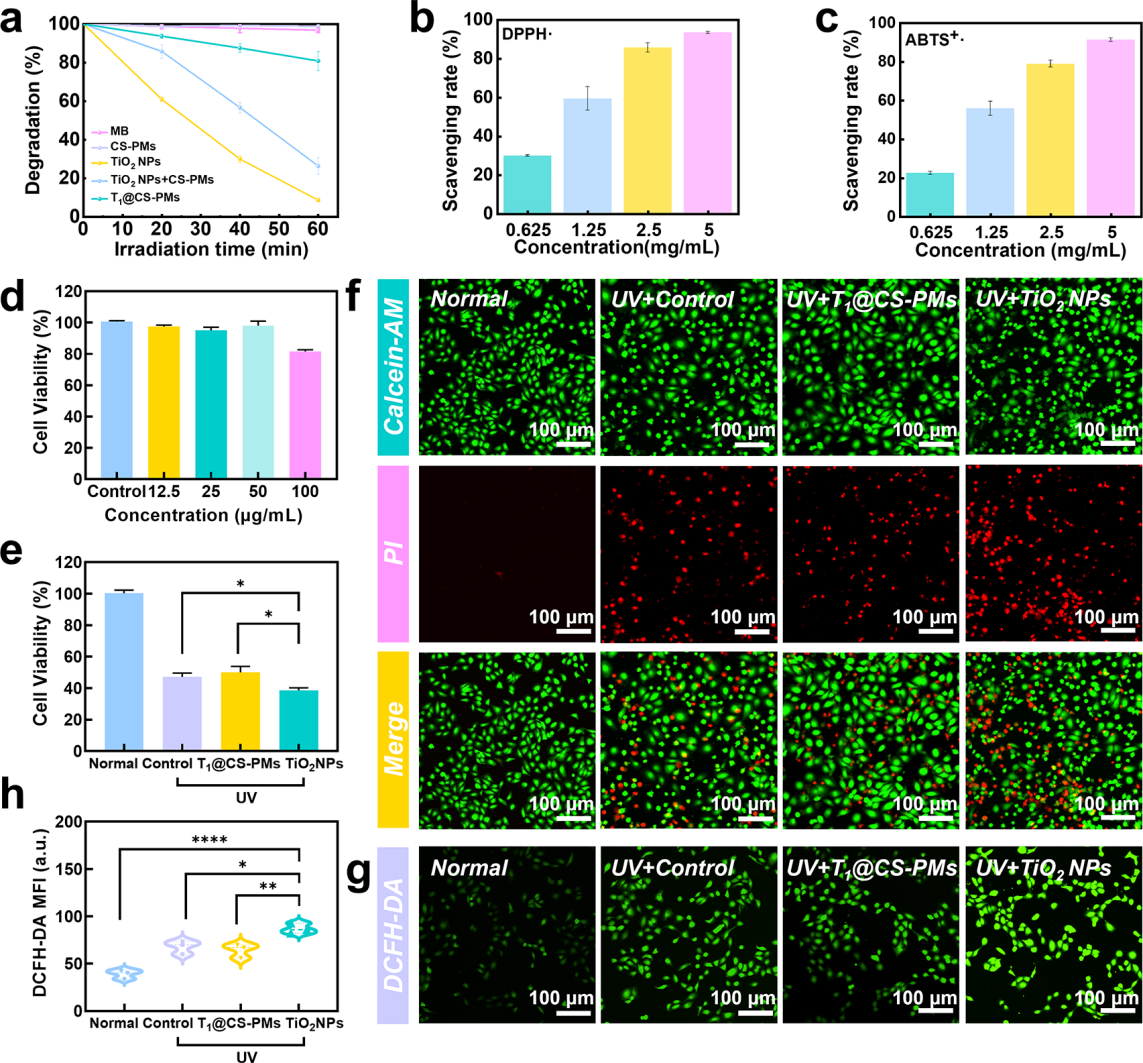
(a) Photocatalytic degradation of MB under UV irradiation with different samples. (b) DPPH• and (c) ABTS^+^• scavenging rates of T_1_@CS-PMs at different concentrations. (d) Viability of HaCaT cells incubated with T_1_@CS-PMs after 24 h. (e) Cell viability, (f) CLSM images of double-stained HaCaT cells with Calcein-AM and PI, (g) CLSM images of intracellular ROS levels, and (h) corresponding fluorescence intensity analysis in HaCaT cells after treatment with different conditions under UV irradiation.

### 3.3 Cytotoxicity and Phototoxicity Assessment of TiO_2_@CS-PMs

The safety of T_1_@CS-PMs was then evaluated using HaCaT cells, a relevant in vitro model for epidermal exposure to sunscreens. First, the intrinsic cytotoxicity of T_1_@CS-PMs was assessed using the CCK-8 assay. Figure 3d reveals that T_1_@CS-PMs demonstrated good biocompatibility, with cell survival exceeding 80% even at a dosage of 100 μg/mL. Given that photoinduced cytotoxicity is a major safety concern when using TiO_2_ NPs as UV filters, we further investigated cell viability under UV irradiation. As shown in Figure 3e, cells treated with TiO_2_ NPs had a significant decline in viability (38.62%). Notably, the T_1_@CS-PMs-treated group showed significantly higher cell viability (50.00%) than the TiO_2_ NPs group and somewhat more than the control group (47.11%) with only UV exposure. Live/dead staining assays (Figure 3f) produced consistent results, with the TiO_2_ NPs group displaying the most intense red fluorescence (PI), indicating significant cell death, whereas the T_1_@CS-PMs and the control group exhibited far fewer dead cells.

To better understand phototoxicity mitigation, intracellular ROS levels were examined with DCFH-DA fluorescent probe (Figure 3g and h). The normal group showed only mild basal fluorescence, indicating very minimal intracellular oxidative stress under healthy settings. After UV irradiation, however, the control group had a significantly increased fluorescence intensity, indicating UV-induced ROS generation. A further increase in fluorescence was seen in the group containing TiO_2_ NPs, indicating excessive ROS accumulation produced by the photocatalytic activity of TiO_2_ NPs after UV exposure. In contrast, encapsulating TiO_2_ NPs within CS-based porous microspheres markedly reduced intracellular ROS generation. T_1_@CS-PMs reduced fluorescence intensity by approximately 26% when compared to TiO_2_ NPs under the same radiation conditions, demonstrating effective mitigation of TiO_2_ NPs-induced oxidative stress amplification. Furthermore, the fluorescence intensity was also slightly lower than that of the control group. These phenomena indicate that the porous CS skeleton effectively lowers photoinduced oxidative stress mediated by TiO_2_ NPs, which is consistent with structure-confined ROS formation. This demonstrates structural confinement as an effective strategy for increasing the safety of inorganic UV filters.

### 3.4 UV-Shielding Property of TiO_2_@CS-PMs and Related Sunscreen

To evaluate the potential of TiO_2_@CS-PMs as UV filters, their optical properties were first investigated. As shown in Figure 4a, TiO_2_ NPs showed low transmittance in the UVB range (280–320 nm), indicating a high intrinsic UV absorption capability. In contrast, CS-PMs had good transmittance over the measuring zone, indicating that the polymeric porous matrix had no substantial contribution to UV shielding. Notably, as compared to TiO_2_ NPs, T_1_@CS-PMs had a wider absorption range that extended into the UVA range, as well as much lower transmittance across both UVB and UVA regions. This increased UV attenuation behavior could be attributed to the porous structure of microspheres, which causes multiple internal light scattering and reflection, increasing the effective optical path length and UV absorption efficiency. To distinguish the contributions of optical confinement and intrinsic electronic structure modulation, the absorption spectra were further analyzed using the Tauc plot method. The calculated optical band gaps for T_1_@CS-PMs and pure TiO_2_ NPs were 2.85 and 3.26 eV, respectively (Figure S12). The reduction of the band gap in T_1_@CS-PMs suggests that interfacial interactions between TiO_2_ NPs and the CS matrix introduce new defect states or surface energy levels, allowing light to be absorbed at longer wavelengths.^48–50^ Overall, the combination of optical confinement within porous microspheres and the band structure change of TiO_2_ NPs leads to better broadband UV shielding. These properties are highly desirable in sunscreens because they could provide effective UV protection with a lower dosage of TiO_2_ NPs.

**Figure 4:**
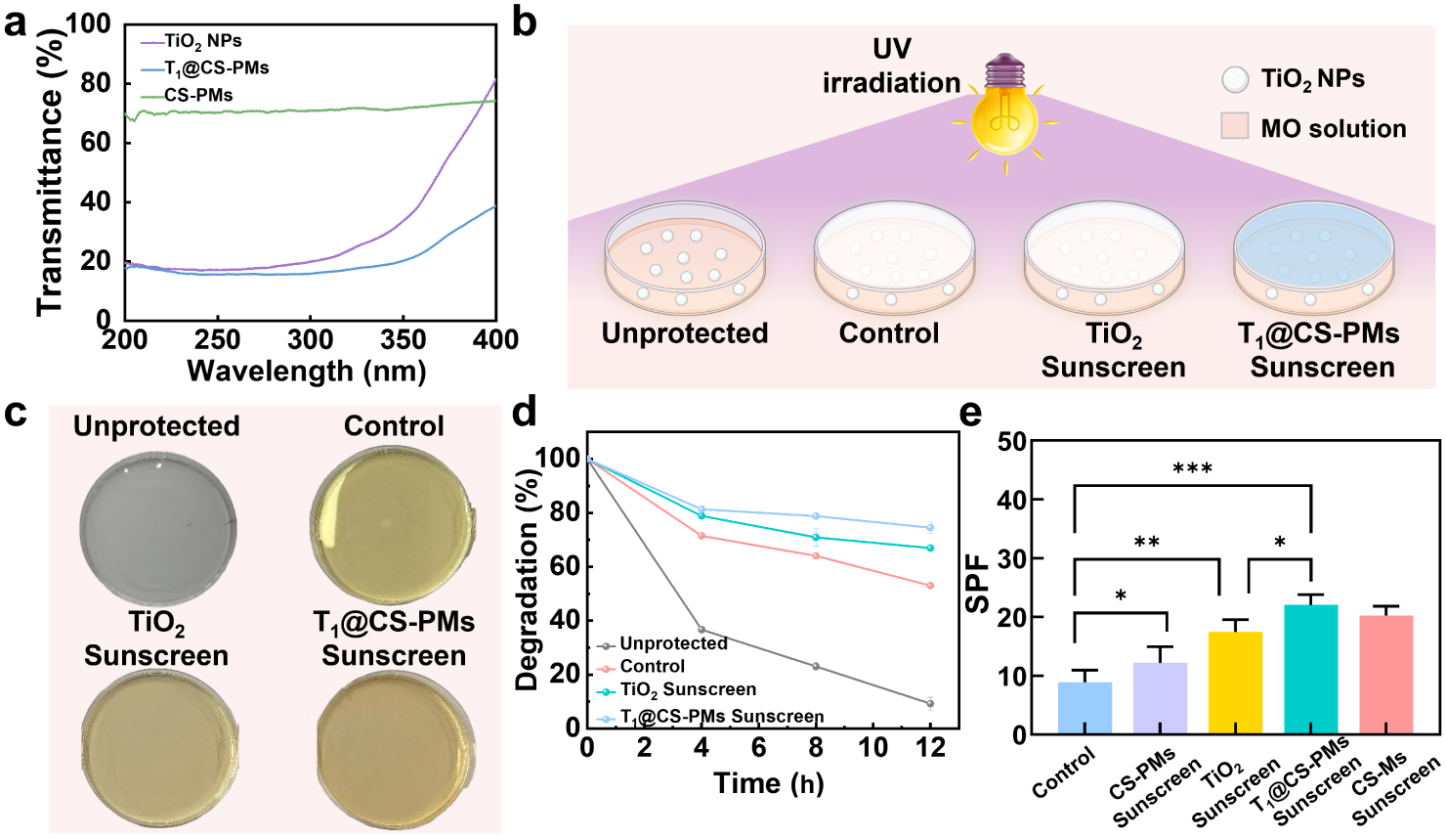
(a) UV–vis transmittance spectra of TiO_2_ NPs, T_1_@CS-PMs, and CS-PMs. (b) Schematic illustration of the in vitro UV-shielding performance of sunscreens using MO solution as a probe. (c) Appearance of MO solutions after 12 h of UV irradiation with or without sunscreen protection. (d) Retention rate of MO solution over time when protected by different sunscreens or not. (e) In vitro SPF values of the different sunscreens.

After examining the safety and optical properties, we investigated the UV-shielding performance of TiO_2_@CS-PMs, which is necessary for their use in sunscreens. The UV-protective effectiveness of different formulations was initially evaluated using UV-induced catalytic degradation of MO as a model. Figure S13 shows the visual appearance of sunscreens containing different particles. The sunscreen containing TiO_2_ NPs (TiO_2_ Sunscreen) had a clear white tint, whereas the sunscreens containing cross-linked CS (CS-Ms, CS-PMs, and T_1_@CS-PMs sunscreen) were cyan. This hue is effective at reducing the whitening impact commonly associated with TiO_2_ NPs. In this test, Petri dish lids coated with sunscreen films were placed over MO solutions containing free TiO_2_ NPs and exposed to UV light. The UV-shielding capability was quantitatively evaluated by monitoring the temporal evolution of the MO retention rate (Figure 4d) and the resulting color change in the solution (Figure 4c). After 12 h of UV irradiation, the unprotected group displayed substantial degradation, with a retention rate of only 9.25% and almost complete decoloration. In comparison, the control group provided only partial protection, with an MO retention rate of 53.00%. The TiO_2_ Sunscreen provided higher UV protection, with a retention rate of 66.95%. Notably, the T_1_@CS-PMs Sunscreen had the best UV protection efficiency, with an MO retention rate of 74.47%. This superior UV-shielding capability is attributed to the synergistic effect of the porous microsphere structure and TiO_2_ NPs, in which the porous architecture promotes light scattering and reflection, while TiO_2_ NPs provide strong intrinsic UV absorption. In addition, SEM confirmed that the T_1_@CS-PMs were uniformly dispersed throughout the sunscreen formulation, which also contributes to the high UV-shielding efficiency (Figure S14).

To convert the intrinsic UV-shielding capability into practical sunscreen efficacy, the SPF values of various formulations were evaluated (Figure 4e). The control group had a low SPF value of 8.90; however, the inclusion of CS-PMs increased the SPF value to 12.18, indicating intrinsic UV attenuation by the porous polymer microspheres. The TiO_2_ Sunscreen exhibited an SPF value of 17.46, demonstrating the good UV shielding of TiO_2_ NPs. Remarkably, the T_1_@CS-PMs Sunscreen achieved the highest SPF value of 22.03, demonstrating the importance of the porous microsphere design in increasing UV screening efficiency. To test the role of microsphere structure, nonporous CS-Ms templated from a water-in-oil Pickering emulsion with a diameter roughly half that of their porous counterparts were prepared as a control (Figure S15). Although nonporous microspheres had a larger BET surface area (60.4 m^2^/g) than porous ones (37.0 m^2^/g), CS-Ms Sunscreens had a lower SPF value. This observation suggests that the porous design has a greater impact on light scattering and reflection than just increasing the surface area. Furthermore, increasing TiO_2_ NP loading (T_3_@CS-PMs and T_5_@CS-PMs Sunscreens) resulted in higher SPF values of 29.74 and 31.84, respectively (Figure S16), suggesting that TiO_2_ NPs content in microspheres can be used to simply and efficiently adjust sunscreen performance. Beyond UV-shielding efficiency, evaluating the photostability of sunscreen is essential for topical applications. T_1_@CS-PMs Sunscreen was exposed to continuous simulated solar light for 8 h, during which transmittance and SPF values were monitored (Figure S17). The UV-shielding efficiency only slightly decreased over the full irradiation period, indicating that T_1_@CS-PMs Sunscreen exhibited good photostability.

### 3.5 Sunscreen Incorporating TiO_2_@CS-PMs for Cell Protection from UV Irradiation

UV-induced DNA damage is a major source of cellular apoptosis and plays a crucial role in the development of skin malignancies. HaCaT cells were selected as a model to evaluate the photoprotective effects of T_1_@CS-PMs Sunscreen. As shown in Figure 5b, UV irradiation without any protection dramatically reduced cell viability to 46.74% when compared to the healthy cells (Normal), indicating severe phototoxicity in the absence of protection. In comparison, the TiO_2_ Sunscreen raised viability to 87.20%. Notably, the T_1_@CS-PMs Sunscreen provided the highest protection, with a cell survival rate of 90.80%. These results indicate that the porous microsphere-based sunscreen is more effective in decreasing UV-induced cytotoxicity. The Calcein-AM/PI double-staining experiment also supported these findings (Figure 5c). Unprotected cells displayed distinct morphological changes, such as cell rounding, increased debris, and a high concentration of red-fluorescent dead cells. Cells protected with TiO_2_ NPs and T_1_@CS-PMs Sunscreens, on the other hand, kept their normal shape and primarily green fluorescence, indicating good cell viability and effective UV-induced apoptosis inhibition. As a result, these results demonstrate that the T_1_@CS-PMs Sunscreen provides strong cellular photoprotection by efficiently blocking UV irradiation and reducing phototoxic damage.

**Figure 5:**
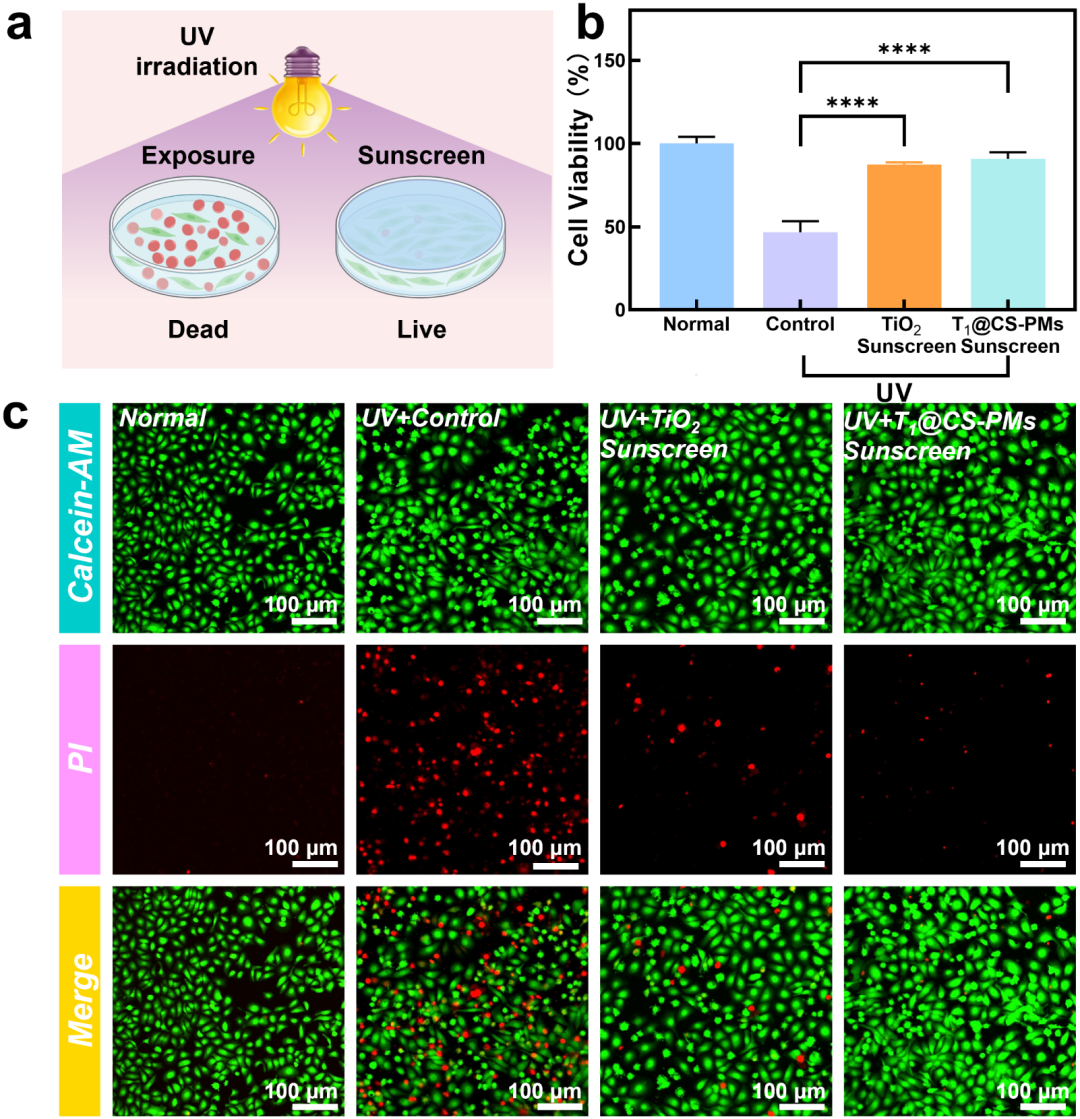
(a) Schematic illustration of the protective effect of sunscreen on HaCaT cells against UV irradiation. (b) Cell viability and (c) CLSM images of double-stained HaCaT cells with Calcein-AM (green, live) and PI (red, dead) after UV irradiation with or without sunscreen protection.

### 3.6 Antibacterial Property of TiO_2_@CS-PMs

An ideal sunscreen should not only provide effective UV protection with minimal phototoxicity but also have microbial safety throughout storage and frequent use. Microbial contamination is a major concern for cosmetic formulations since it compromises product stability and can cause skin diseases. CS is well-known for its intrinsic antibacterial activity, suggesting that TiO_2_@CS-PMs may provide additional antimicrobial functionality. Therefore, the antibacterial effectiveness of the as-prepared TiO_2_@CS-PMs was evaluated. Figure 6 shows a quantitative assessment of antibacterial activity of T_1_@CS-PMs against *Staphylococcus aureus* (Gram-positive) and *Escherichia coli* (Gram-negative) using a standard colony-counting method. A dose-dependent reduction in bacterial colonies was observed, demonstrating antibacterial efficacy. At a dose of 20 mg/mL, the inhibition rates for *S. aureus* and *E. coli* were around 90% and 78%, respectively. These results demonstrate that the T_1_@CS-PMs have broad-spectrum antibacterial action and a higher susceptibility to Gram-positive bacteria.

**Figure 6:**
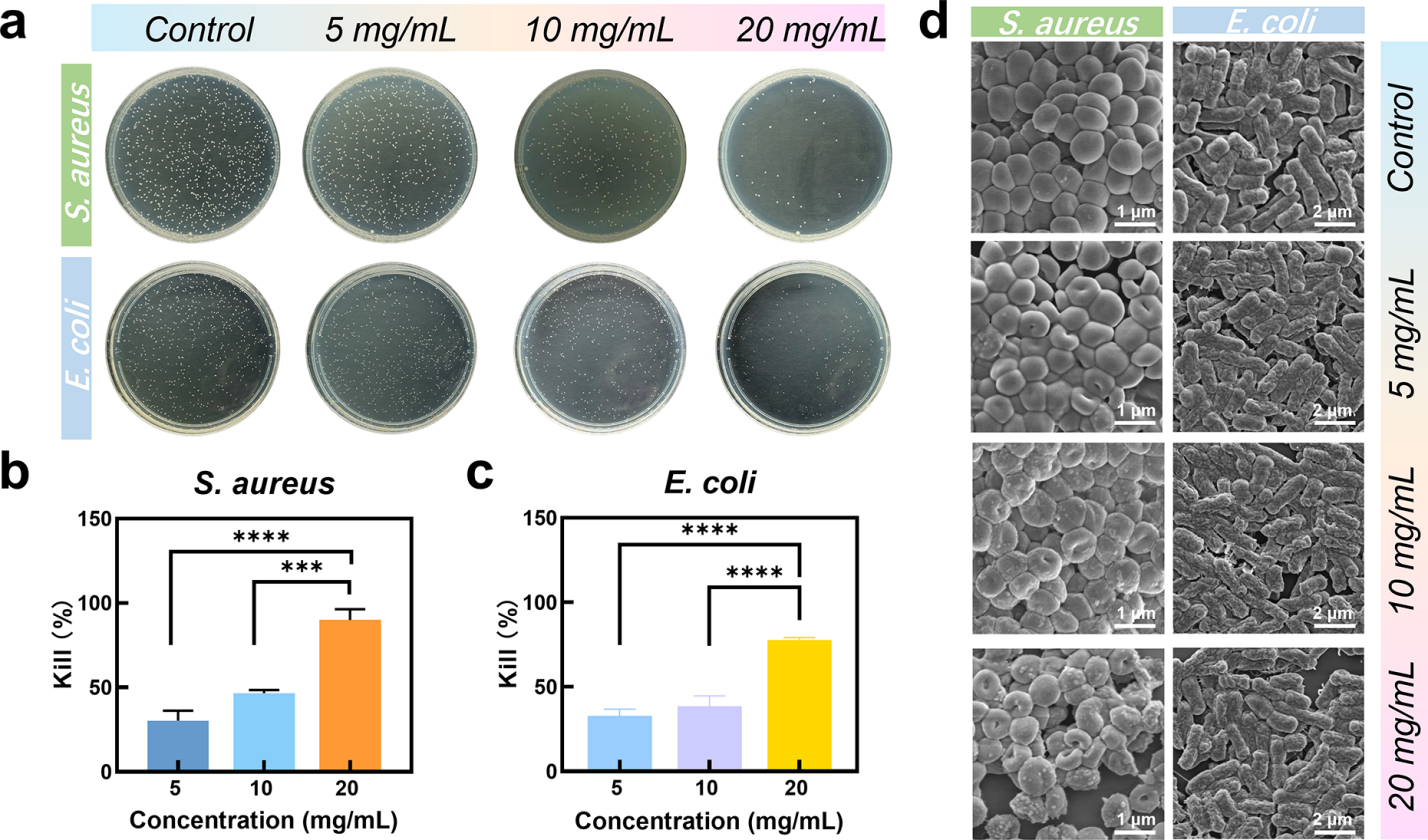
(a) Appearances of the bacterial colonies of *S. aureus* and *E. coli* incubated with different concentrations of T_1_@CS-PMs; bactericidal percentage of T_1_@CS-PMs at different concentrations against (b) *S. aureus* and (c) *E. coli*; (d) SEM images of *S. aureus* and *E. coli* without or with T_1_@CS-PMs treatment.

The antibacterial mechanism was next examined using SEM (Figure 6d). In the control groups, *S. aureus* and *E. coli* retained their spherical and rod-shaped morphologies. T_1_@CS-PMs, however, caused substantial morphological damage in bacteria. *S. aureus* cells collapsed and deformed, indicating peptidoglycan network rupture and intracellular component leakage.^48,49^ For *E. coli*, T_1_@CS-PMs interacted with negatively charged components on the outer membrane via electrostatic attraction, increasing membrane permeability, impairing nutrient transport, and causing intracellular osmotic imbalance, ultimately resulting in bacterial death.^51,52^ In addition, the antibacterial performance of CS-PMs under identical conditions was also evaluated (Figure S18). These showed concentration-dependent antibacterial activity, with inhibition rates similar to those of T_1_@CS-PMs. These findings demonstrate that TiO_2_@CS-PMs have natural antibacterial properties derived from the CS matrix, providing an additional safety benefit for cosmetic applications by preventing microbial contamination without the need of conventional preservatives.

## 4 Conclusion

In summary, we demonstrated that the Pickering double emulsion templating strategy is an effective route to prepare CS-based porous microspheres encapsulating TiO_2_ NPs with adjustable composition and microstructure. The encapsulation of TiO_2_ NPs in the porous polymeric skeleton dramatically inhibited their photocatalytic activity and ROS formation under UV irradiation. Consequently, the as-prepared TiO_2_@CS-PMs demonstrated good biocompatibility and considerably lower phototoxicity than naked TiO_2_ NPs during UV exposure. Furthermore, these microspheres had broadened and improved UV attenuation owing to the porous architecture and interfacial interactions between TiO_2_ NPs and the CS matrix. Sunscreen incorporating TiO_2_@CS-PMs showed increased UV-shielding effectiveness and SPF value at lower TiO_2_ NPs doses, as well as improved cellular photoprotection. Additionally, these microspheres demonstrated antibacterial activity, with concentration-dependent bactericidal performance and higher efficacy against *S. aureus*. Overall, the prepared porous microspheres represent a promising platform toward safer and multifunctional sunscreens by integrating suppressed photocatalytic activity, excellent photoprotection, and additional antibacterial activity.

## Supplementary Material



## Notes

Three-dimensional CLSM reconstruction of TiO_2_@CS-PMs; optical microscopy images, SEM images, and size distributions of O/W/O Pickering double emulsions and the corresponding porous microspheres prepared under different oil/water volume ratios, chitosan concentrations, and TiO_2_ NPs feed amounts; TEM images and size distributions of pristine TiO_2_ and SiO_2_ NPs; thermogravimetric analysis of T_3_@CS-PMs and T_5_@CS-PMs; UV-induced degradation experiment; contact angle measurements, Tauc plots, and band gap analysis of TiO_2_ NPs and T_1_@CS-PMs; digital image of various sunscreen formulations; SEM image of T_1_@CS-PMs incorporated into a commercial cream; optical microscopy image, SEM image, and size distributions of nonporous CS microspheres; and in vitro SPF values of T_3_@CS-PMs and T_5_@CS-PMs Sunscreens; UV transmittance and SPF value of T_1_@CS-PMs Sunscreen during simulated solar light irradiation; antibacterial property of CS-PMs against *S. aureus* and *E. coli* (PDF)
