# Supplementary material for: Encapsulating TiO2 Nanoparticles in Chitosan-Based Porous Microspheres as Multifunctional UV Filters
Source: Langmuir. 2026 Jun 5;42(23):16621–32. doi: 10.1021/acs.langmuir.6c01320 (PMC13276904; doi:10.1021/acs.langmuir.6c01320)
Supplement: Supplementary file 1 [file la6c01320_si_001.pdf]

Supporting Information

Encapsulating TiO_2 nanoparticles in chitosan-based porous microspheres as multifunctional UV filters

Yunxing Li*†, Xue Cai†, Beizhe Chang†, Tong Yang†, Hang Jiang†, To Ngai*‡

† *Key Laboratory of Synthetic and Biological Colloids, Ministry of Education, School of Chemical and Material Engineering, Jiangnan University, Wuxi 214122, China.*

‡ *Department of Chemistry, The Chinese University of Hong Kong, Hong Kong 999077, China.*

* Corresponding author

E-mail: yunxingli@jiangnan.edu.cn; tongai@cuhk.edu.hk

Number of pages: 11

Number of figures: 18

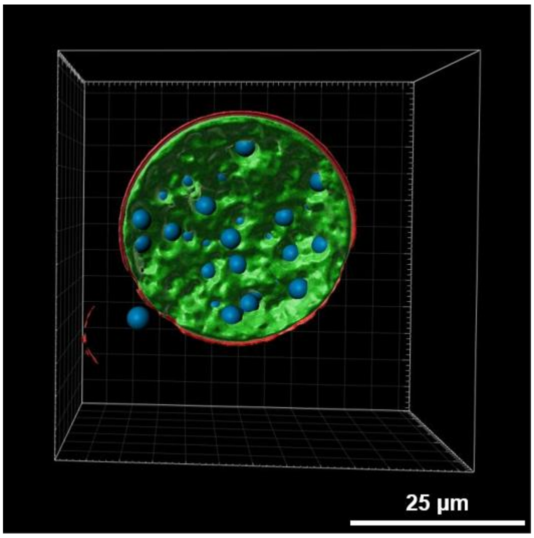

Figure S1. 3D reconstructed confocal image of T_1@CS-PMs with FITC-labeled CS, RhB-labeled SiO_2 NPs, and perylene-labeled oil phase.

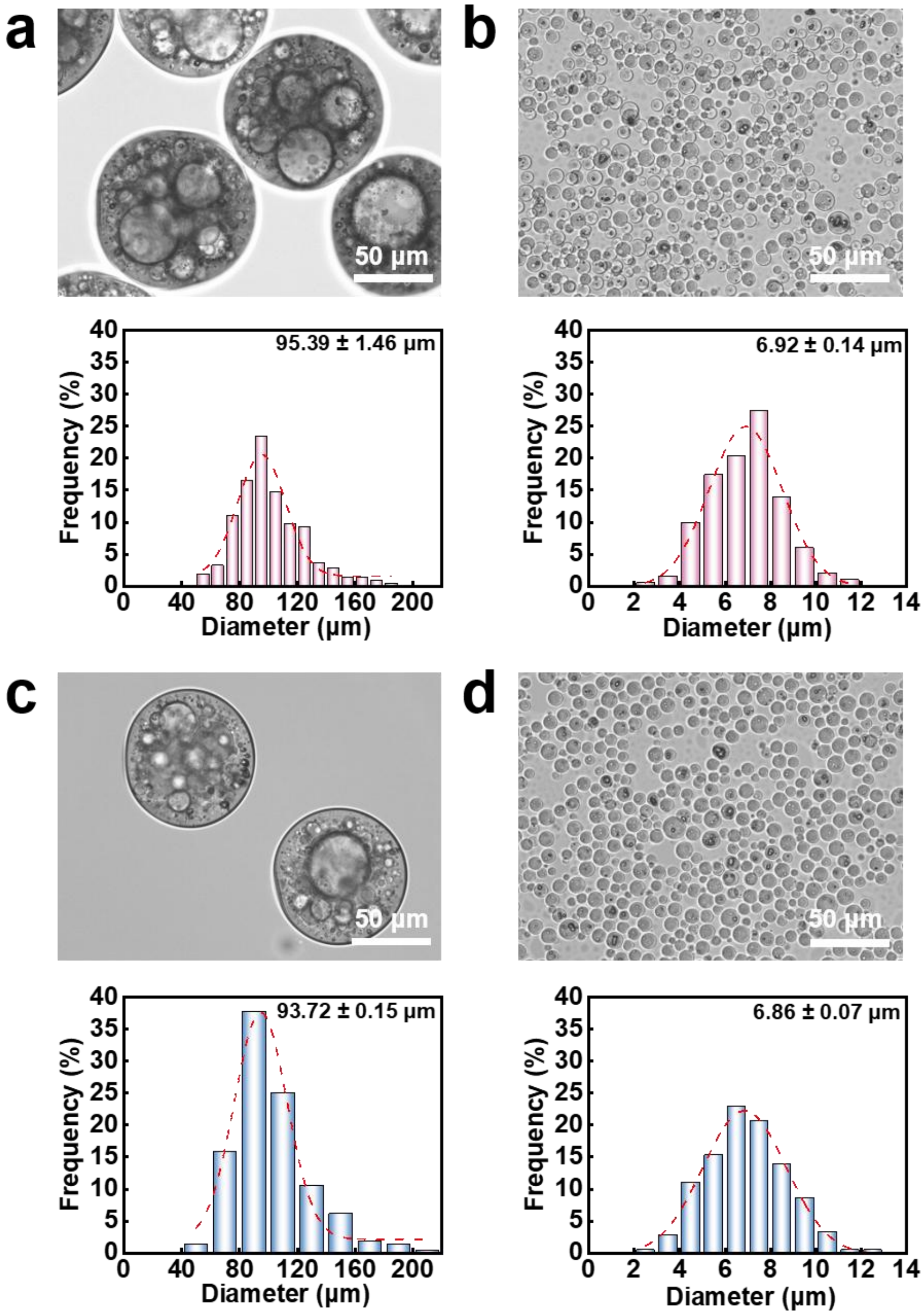

Figure S2. Optical microscopy images and size distributions of O/W/O Pickering emulsions and the corresponding TiO_2@CS-PMs prepared with water/oil volume ratios of 2:1 (a and c) and 1:2 (b and d), respectively.

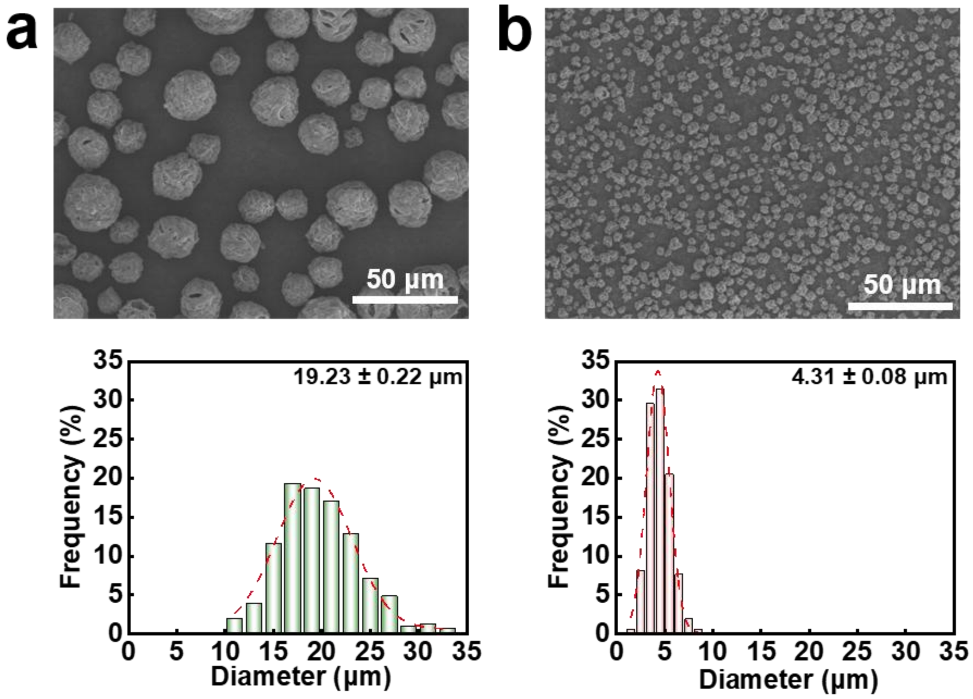

Figure S3. SEM images and size distributions of TiO_2@CS-PMs prepared with water/oil volume ratios of 2:1 (a) and 1:2 (b).

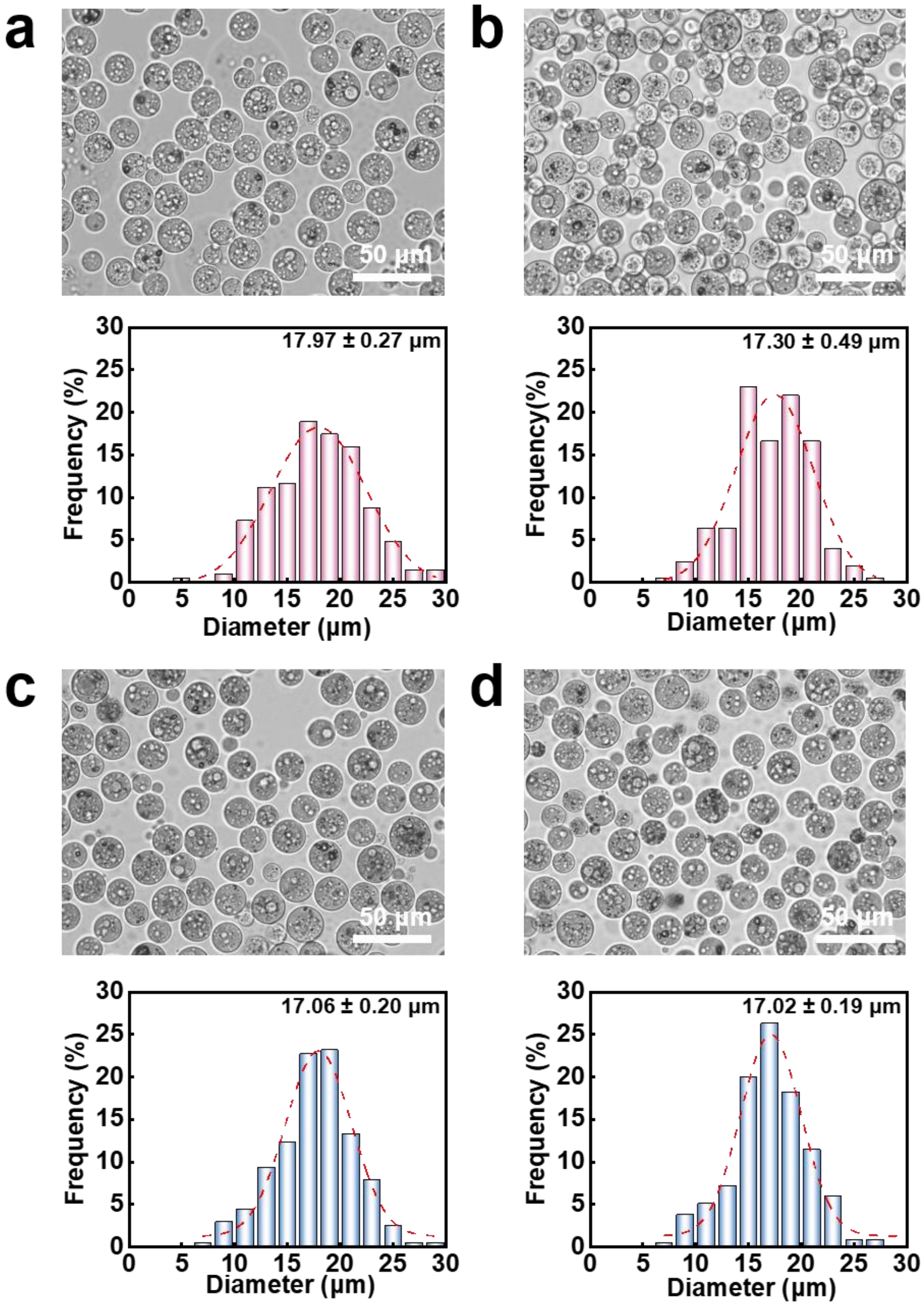

Figure S4. Optical microscopy images and size distributions of O/W/O Pickering emulsions and the corresponding TiO_2@CS-PMs prepared with CS concentrations of 2% (a and c) and 2.5% (b and d), respectively.

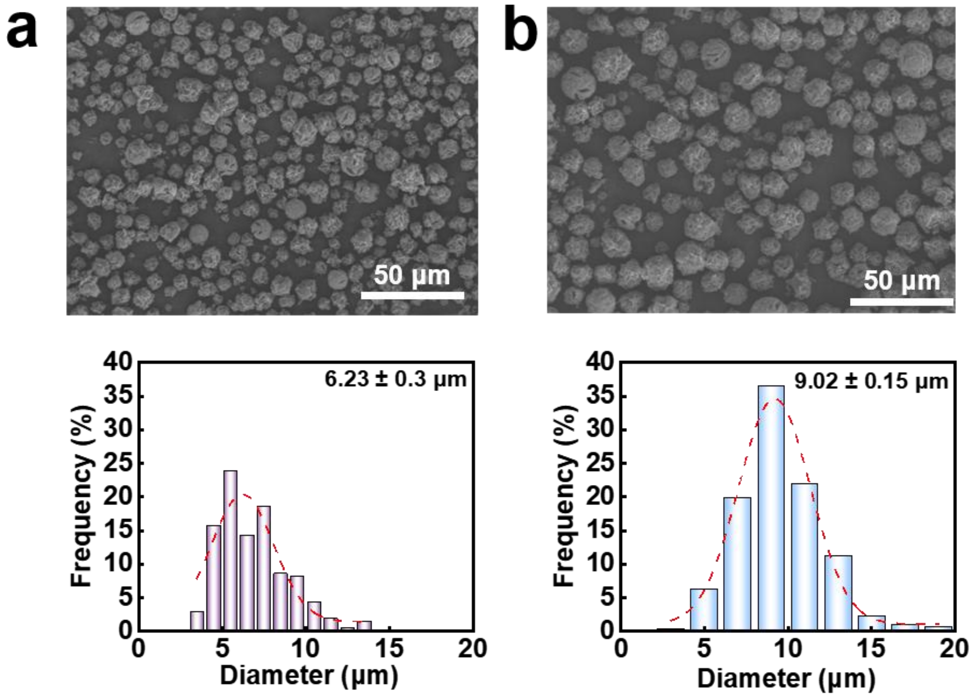

Figure S5. SEM images and size distributions of TiO_2@CS-PMs prepared with different CS concentrations: (a) 2.0% and (b) 2.5% (w/v).

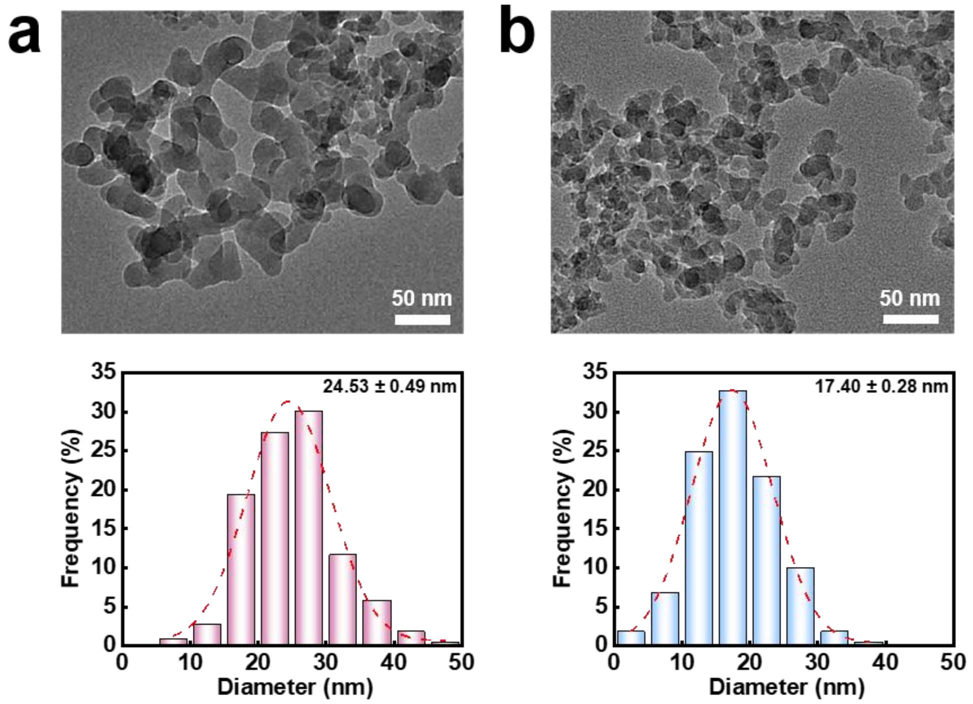

Figure S6. TEM images and size distributions of TiO_2 NPs (a) and SiO_2 NPs (b).

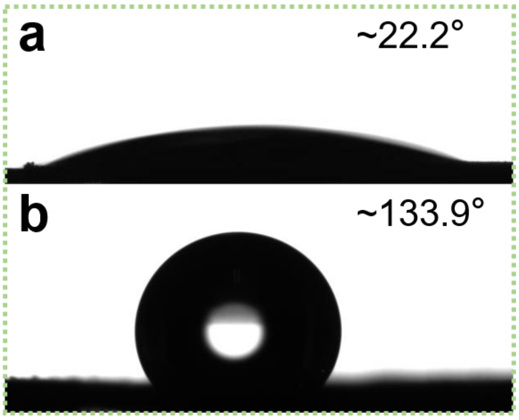

Figure S7. Contact angle of water droplets in air resting on the substrates consisting of TiO_2 NPs (a) and T_1@CS-PMs (b).

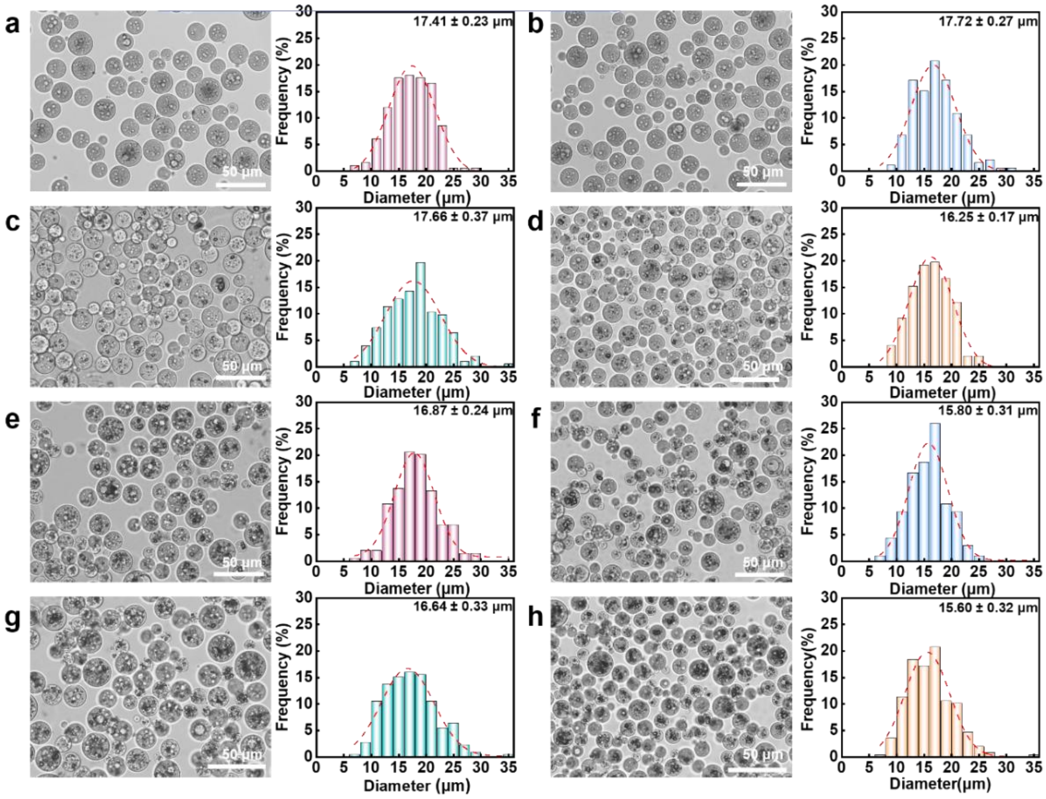

Figure S8. Optical microscopy images and size distributions of O/W/O Pickering double emulsions and the corresponding CS-PMs and TiO_2@CS-PMs prepared with varying TiO_2 NPs feed amounts: (a and b) 0, (c and d) 30, (e and f) 90, and (g and h) 150 mg, respectively.

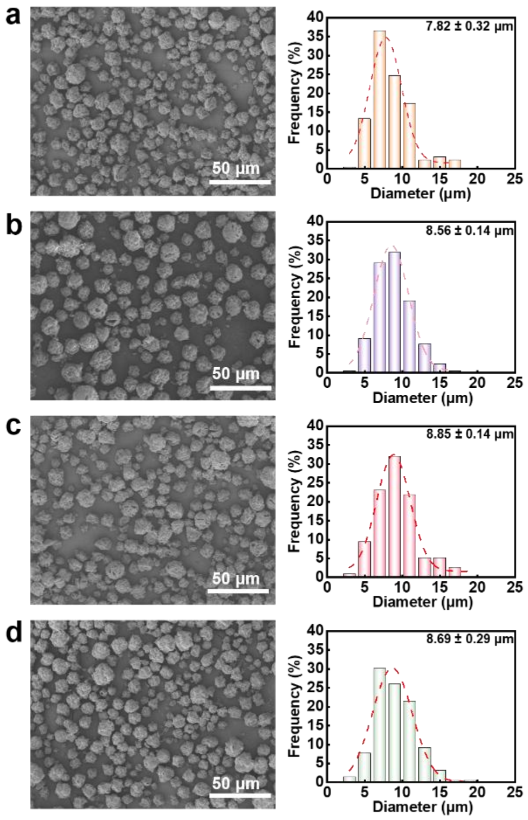

Figure S9. SEM images and size distribution of the prepared microspheres: (a) CS-PMs, (b) T_1@CS-PMs, (c) T_3CS-PMs, and (d) T_5@CS-PMs.

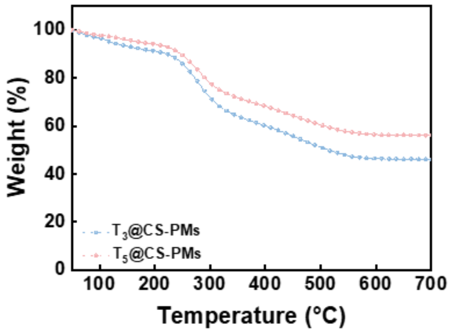

Figure S10. TGA curves of T_3@CS-PMs and T_5@CS-PMs.

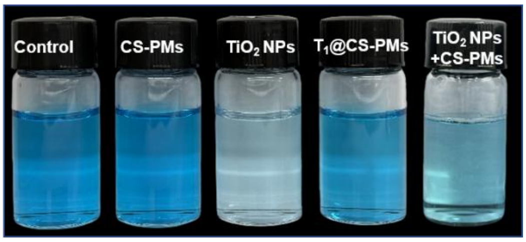

Figure S11. Digital image of MB solutions (control, no particles) and MB solutions with different particles after 1 h of UV irradiation.

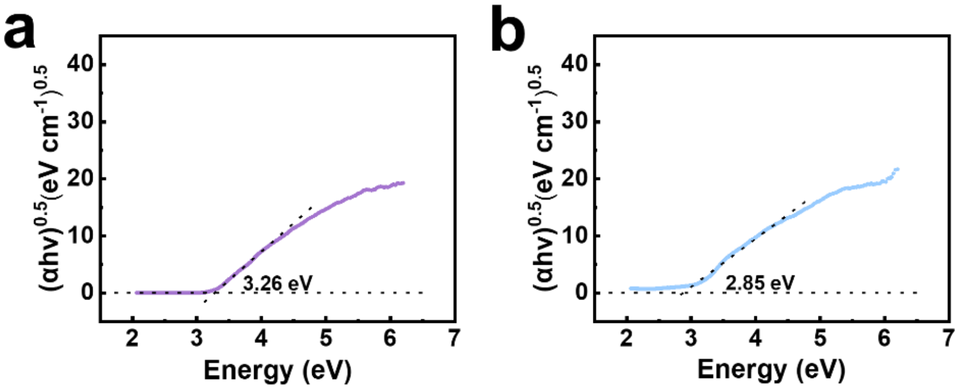

Figure S12. Tauc plots and related band gap values of (a) TiO_2 NPs and (b) T_1@CS-PMs.

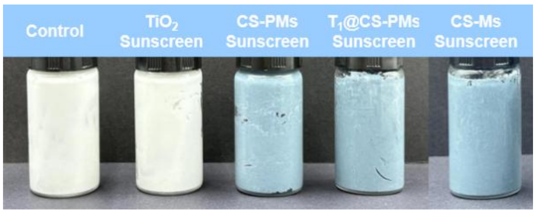

Figure S13. Appearance of various sunscreens.

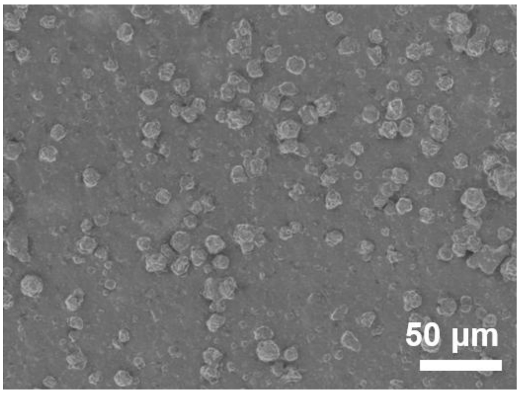

Figure S14. SEM image of T_1@CS-PMs incorporated into a commercial cream.

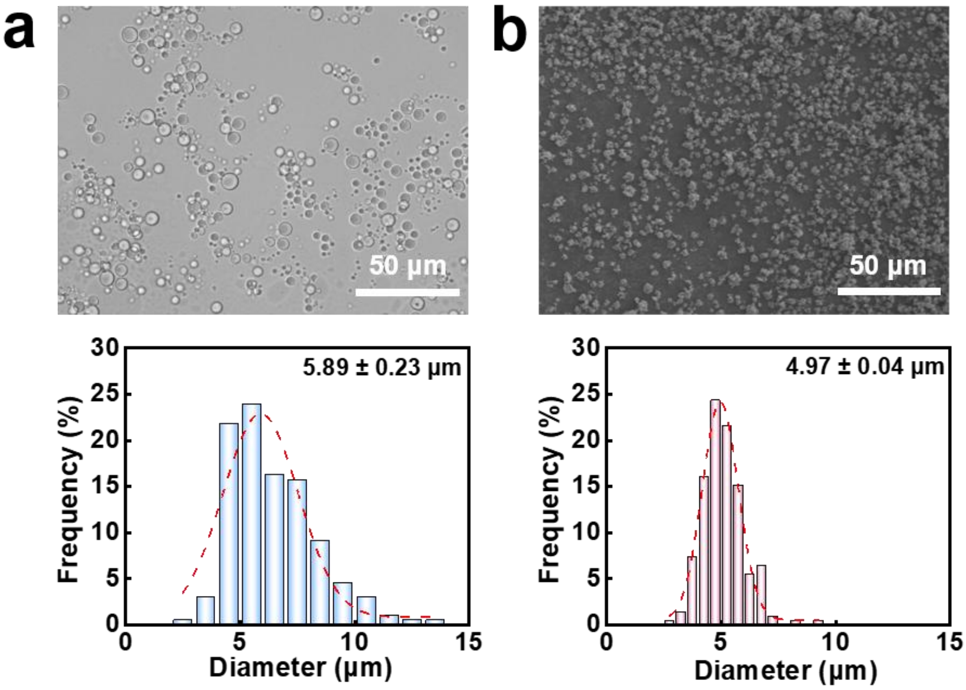

Figure S15. (a) Optical microscopy images and size distributions of the W/O Pickering emulsion with water/oil volume ratio of 1:4 after CS crosslinking. (b) SEM image and size distribution of the corresponding chitosan-based microspheres (CS-MS).

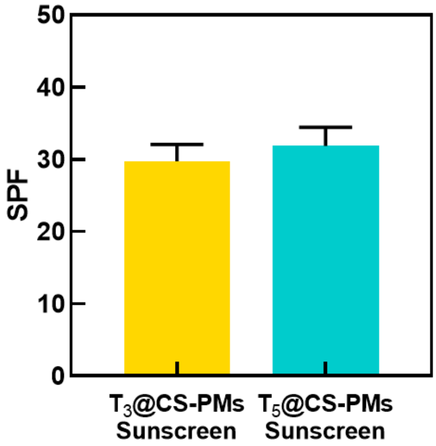

Figure S16. In vitro SPF values of T_3@CS-PMs and T_5@CS-PMs Sunscreens.

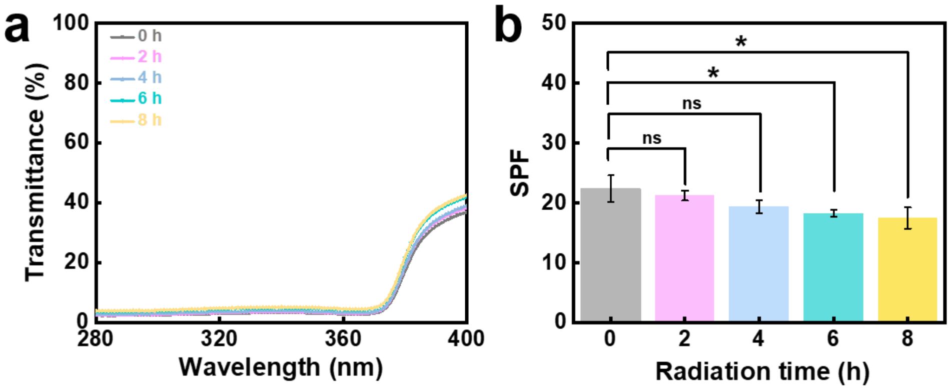

Figure S17. (a) UV transmittance and (b) SPF values of T_1@CS-PMs Sunscreen during 8 h of continuous simulated solar irradiation.

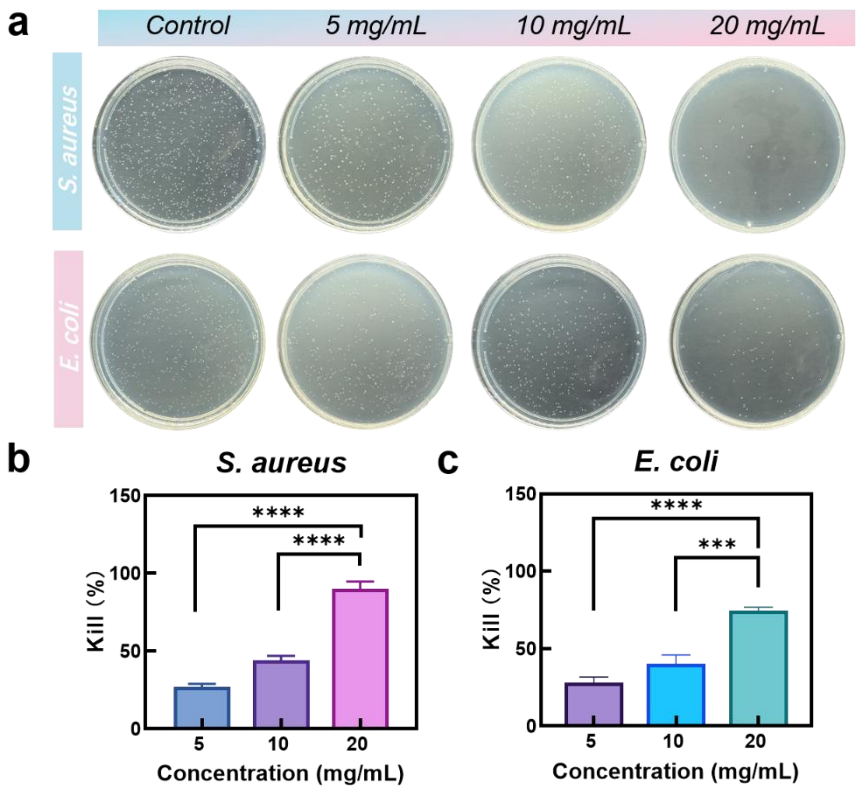

Figure 18. (a) Appearances of the bacteria colonies of *S. aureus* and *E. coli* incubated with different concentrations of CS-PMs; bactericidal percentage of CS-PMs at different concentrations against (b) *S. aureus* and (c) *E. coli*.
